# Rapamycin, by Inhibiting mTORC1 Signaling, Prevents the Loss of Striatal Bidirectional Synaptic Plasticity in a Rat Model of L-DOPA-Induced Dyskinesia

**DOI:** 10.3389/fnagi.2020.00230

**Published:** 2020-08-11

**Authors:** Valeria Calabrese, Anna Di Maio, Gioia Marino, Antonella Cardinale, Giuseppina Natale, Arianna De Rosa, Federica Campanelli, Maria Mancini, Francesco Napolitano, Luigi Avallone, Paolo Calabresi, Alessandro Usiello, Veronica Ghiglieri, Barbara Picconi

**Affiliations:** ^1^Laboratory of Experimental Neurophysiology, IRCCS San Raffaele Pisana, Rome, Italy; ^2^Department of Medicine, University of Perugia, Perugia, Italy; ^3^CEINGE Biotecnologie Avanzate, Naples, Italy; ^4^Laboratory of Neurophysiology, IRCCS Santa Lucia Foundation, Rome, Italy; ^5^Department of Experimental Medicine, Sapienza University of Rome, Rome, Italy; ^6^Department of Veterinary Medicine and Animal Productions, University of Naples Federico II, Naples, Italy; ^7^Neurologia, Fondazione Policlinico Universitario Agostino Gemelli IRCCS, Rome, Italy; ^8^Dipartimento di Neuroscienze, Università Cattolica del Sacro Cuore, Rome, Italy; ^9^Department of Environmental, Biological and Pharmaceutical Sciences and Technologies (DISTABIF), University of Campania Luigi Vanvitelli, Caserta, Italy; ^10^Università Telematica San Raffaele, Rome, Italy

**Keywords:** depotentiation, functional plasticity, levodopa-induced dyskinesia, Parkinson’s disease, dorsolateral striatum

## Abstract

Levodopa (L-DOPA) treatment is the main gold-standard therapy for Parkinson disease (PD). Besides good antiparkinsonian effects, prolonged use of this drug is associated to the development of involuntary movements known as L-DOPA-induced dyskinesia (LID). L-DOPA-induced dyskinesia is linked to a sensitization of dopamine (DA) D1 receptors located on spiny projection neurons (SPNs) of the dorsal striatum. Several evidences have shown that the emergence of LID can be related to striatal D1/cAMP/PKA/DARPP-32 and extracellular signal-regulated kinases (ERK1/2) pathway overactivation associated to aberrant *N-methyl-d-aspartate (NMDA)* receptor function. In addition, within striatum, ERK1/2 is also able to modulate in a D1 receptor-dependent manner the activity of the mammalian target of rapamycin complex 1 (mTORC1) pathway under DA depletion and L-DOPA therapy. Consistently, increased mTORC1 signaling appears during chronic administration of L-DOPA and shows a high correlation with the severity of dyskinesia. Furthermore, the abnormal activation of the D1/PKA/DARPP-32 cascade is paralleled by increased phosphorylation of the GluA1 subunit of the α-amino-3-hydroxy-5-methyl-4-isoxazolepropionic acid (AMPA) receptor at the PKA Ser845 site. The GluA1 promotes excitatory AMPA receptor-mediated transmission and may be implicated in the alterations found at the corticostriatal synapses of dyskinetic animals. In our study, we investigated the role of mTORC1 pathway activation in modulating bidirectional striatal synaptic plasticity in L-DOPA-treated parkinsonian rats. Inhibition of mTORC1 by coadministration of rapamycin to L-DOPA was able to limit the magnitude of LID expression, accounting for a therapeutic effect of this drug. In particular, behavioral data showed that, in L-DOPA-treated rats, rapamycin administration induced a selective decrease of distinct components of abnormal involuntary movements (i.e., axial and orolingual dyskinesia). Furthermore, *ex vivo* patch clamp and intracellular recordings of SPNs revealed that pharmacological inhibition of mTORC1 also resulted associated with a physiological bidirectional plasticity, when compared to dyskinetic rats treated with L-DOPA alone. This study uncovers the important role of mTORC1 inhibition to prevent the loss of striatal bidirectional plasticity under chronic L-DOPA treatment in rodent models of PD.

## Introduction

Parkinson disease (PD) is a progressive neurodegenerative disorder, in which the severe degeneration of substantia nigra pars compacta (SNc) neurons results in the denervation of dopaminergic afferents in the striatum, the main target of nigral fibers (Braak et al., 2003; Wirdefeldt et al., 2011; Postuma et al., 2015). Such extensive decrease of striatal dopamine (DA) levels causes both altered glutamatergic neurotransmission and behavioral deficits, including motor and nonmotor symptoms (Calabresi et al., 1997; Fahn, 2015; Kouli et al., 2018; Picconi et al., 2018a,b). The finely regulated ability to adapt movement control to a given motor task depends on synaptic plasticity mechanisms, which operate on the cortex and the striatum (Calabresi et al., 2014). Synaptic plasticity in cortical and striatal neurons controls the neuronal excitability of basal ganglia nuclei and limits enhanced neuronal activity thus ensuring stability and integrity of surrounding circuits that regulate motor and nonmotor aspects of behavior. The state of neuronal synaptic plasticity allows neurons to detect their working state and to adapt their properties to maintain a correct homeostasis, determining a correlation between environmental events and the ability to store new information (Calabresi et al., 2016). The synaptic efficiency is expressed by the ability of striatal spiny projection neurons (SPNs) to respond to a given stimulation with a long-term potentiation (LTP) or a long-term depression (Calabresi et al., 1992, 2000a; Malenka and Bear, 2004). Accordingly, dysfunctions in these two forms of synaptic plasticity or the loss of synaptic downscaling well correlates with impaired control of voluntary movements (Calabresi et al., 2016).

The most used therapeutic approach to alleviate parkinsonian motor symptoms is the administration of DA precursor, levodopa (L-DOPA; Nutt, 1999; Fahn, 2000, 2015; Obeso et al., 2000, 2017; Olanow and Schapira, 2013; Espay et al., 2018). The striatum is considered the main target of L-DOPA, which efficiently provides a restoration of the concentrations of DA (Cotzias et al., 1967; Birkmayer and Hornykiewicz, 1998; Fahn, 2015; Zahoor et al., 2018). Besides its antiparkinsonian effects, however, long-term use of L-DOPA causes, in most patients, motor fluctuations and involuntary hyperkinetic movements (i.e., dyskinesia; Calabresi et al., 2010). Development of L-DOPA-induced dyskinesia (LID) in PD patients and in experimental animal models is associated to defective bidirectional plasticity that primarily manifests with the loss of synaptic depotentiation (Picconi et al., 2003; Bastide et al., 2015). In laboratory rats, DA deprivation induced by the injection of 6-hydroxydopamine (6-OHDA) in the medial forebrain bundle (MFB) causes loss of corticostriatal LTP (Centonze et al., 1999; Picconi et al., 2003). This form of synaptic plasticity is restored by L-DOPA treatment. However, chronic L-DOPA administration in parkinsonian rats also triggers dyskinetic behavior (Picconi et al., 2003), along a complete absence of synaptic depotentiation, a form of metaplasticity that can be normally observed in healthy rats by the application of a low-frequency stimulation (LFS) protocol after LTP induction. The mechanism underlying the loss of this form of homeostatic plasticity was found to be associated with increased activity of the molecular signaling cascade downstream DA D1 receptor (D1R) activation (Picconi et al., 2003), whose hyperactivity has a key role in LID induction (Aubert et al., 2005; Santini et al., 2007; Lindgren et al., 2010; Feyder et al., 2011). In this regard, the emergence of LID in rodents is known to depend on abnormally high D1R-dependent Protein Kinase A (PKA) pathway activation (Calabresi et al., 2000b; Picconi et al., 2003; Santini et al., 2007, 2010, 2012; Lebel et al., 2010), which, in turn, increases the phosphorylation state of its striatal substrate protein DA- and cyclic adenosine monophosphate (cAMP)-regulated phosphoprotein 32 kDa (DARPP-32) at the threonine-34 (Thr34) site.

Besides DARPP-32 activity, cAMP/PKA signaling also affects other proteins in the striatum of DA-depleted rodents treated with L-DOPA. These substrates include *N*-methyl-D-aspartate (NMDA), α-amino-3-hydroxy-5-methyl-4-isoxazolepropionic acid (AMPA) receptors (Chase and Oh, 2000; Santini et al., 2007) and extracellular signal–regulated kinases 1/2 (ERK1/2; Santini et al., 2007).

In this regard, previous studies carried out in 6-OHDA-lesioned mice treated with L-DOPA or D1 agonist drug (SKF81297) highlighted the ability of striatal ERK1/2 signaling to induce mammalian target of rapamycin (mTOR) pathway overactivation (Santini et al., 2007). Mammalian target of rapamycin performs its functions primarily through two complexes: mammalian target of rapamycin complex 1 (mTORC1), involved in the control of protein synthesis and in autophagy, and mTOR complex 2 (mTORC2), implicated in the organization of actin dynamics and in cell survival (Costa-Mattioli et al., 2009; Perluigi et al., 2015). Thus, in addition to its regulatory role on transcription, ERK1/2 is also able to modulate the activity of mTORC1, known to be directly linked to LID (Santini et al., 2009). Furthermore, independent observations (Santini et al., 2009; Brugnoli et al., 2016) demonstrated that administration of the mTORC1 inhibitor rapamycin (Hosoi et al., 1999; Sarbassov et al., 2006) in hemiparkinsonian mice resulted in a robust reduction of LID without interfering with the beneficial effects of L-DOPA treatment. In line with these observations, very recently Subramaniam et al. showed that up-regulation of RASGRP1 in dorsal striatum controlled the magnitude of LID in mice by increasing mTOR signaling (Eshraghi et al., 2020).

Here, we postulated that rapamycin, by inhibiting abnormal striatal mTORC1 activation, would limit the emergence of L-DOPA-induced abnormal involuntary movements (AIMs) through the preservation of bidirectional plasticity of SPNs. To validate this hypothesis, we explored the still unknown electrophysiological and biochemical consequences associated to pharmacological mTORC1 inhibition in hemiparkinsonian rats chronically treated with L-DOPA.

## Materials and Methods

All the experiments were approved by the Institutional Animal Care and Use Committee and performed according to the Guidelines of The European Union Council (2010/6106/UE) and by the Italian Ministry of Health (D.Lgs. 295/2012-A and D.Lgs. 26/2014; prot. 1296/2015 PR).

### 6-Hydroxydopamine Hydrochloride (6-OHDA) Injection and Apomorphine Test

For the unilateral nigrostriatal DA denervation, adult male Wistar rats (*n* = 52; Charles River Laboratories, Calco, Italia) were deeply anesthetized with Zoletil (an anesthetic drug; 50/50 mg/ml tiletamine and zolazepam) and Rompun (a sedative drug; 20 mg/ml xylazine hydrochloride) and were stereotaxically injected with 6-OHDA (3 μg/μl in saline containing 0.1% ascorbic acid, volume injected 4 μl) into the MFB of the left hemisphere using a standard 10 μl Hamilton syringe (Ghiglieri et al., 2016). Coordinates were calculated in mm from the bregma and the dura surface of the rats: tooth bar −2.3, anteroposterior (AP) −4.4 mm; mediolateral (ML) + 1.2 mm, and dorsoventral −7.8 mm at a rate of 0.38 μl/min (Paxinos et al., 1985; Paillé et al., 2010; Bagetta et al., 2012; Ghiglieri et al., 2016).

**Figure 1:**
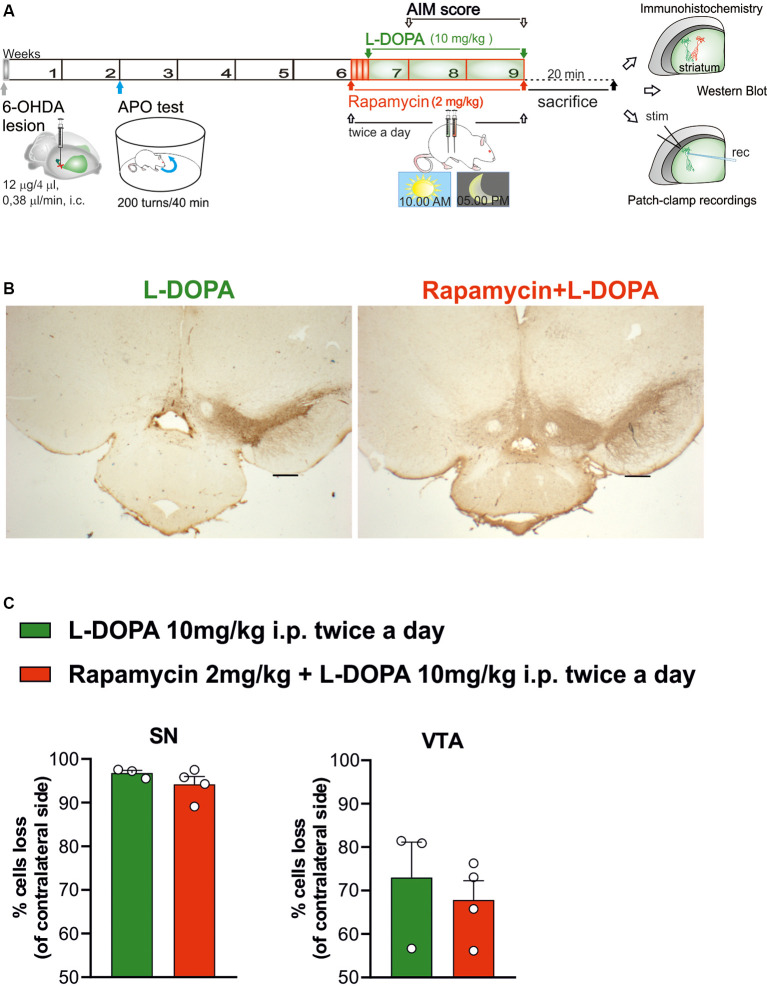
Histochemical and biochemical characterization. **(A)** Schematic experimental protocol. **(B)** Tyrosine hydroxylase immunostaining in coronal section of Substantia nigra pars compacta (SNc) and ventral tegmental area (VTA) regions of 6-hydroxydopamine (6-OHDA)-lesioned rats treated with Levodopa (L-DOPA) or rapamycin + L-DOPA (2× objective, scale bar 500 μm). **(C)** The graphs show the percentage of loss of TH+ cells (compared to contralateral side), in the ipsilateral side of L-DOPA or rapamycin + L-DOPA-treated rats, respectively, in the SNc region (**C**, left panel; *p* = 0.2957) and VTA region (**C**, right panel; *p* = 0.5757). Data were expressed as mean ± SEM and analyzed by a two-tailed *t*-test.

In order to assess the efficacy of the 6-OHDA lesion, 15 days after the surgery, lesioned rats were tested with a subcutaneous injection of apomorphine (0.05 mg/kg; dissolved in in 0.9% NaCl saline), and the turns contralateral to the lesioned side were counted for 40 min using an automatic rotometer (Ungerstedt, 1971; Picconi et al., 2008). Two months after the surgery, 42 fully lesioned rats, showing more than 200 contralateral turns, were subjected to drug treatments, behavioral test, and biochemical and electrophysiological experiments (Figure 1A). This time interval from surgery was to avoid any interference of apomorphine treatment with the DA-dependent plastic changes occurring after dopaminergic denervation (Herrera-Marschitz and Ungerstedt, 1984; Schwarting and Huston, 1996; Picconi et al., 2004).

### Drug Treatments

Experiments were conducted using two experimental groups. One group (*n* = 21 rats) was treated for 2 weeks with only L-DOPA [10 mg/kg L-DOPA plus 6 mg/kg benserazide, intraperitoneal (i.p.) twice a day; dissolved in 0.9% NaCl saline solution]. A second group (*n* = 31 rats) was treated with rapamycin (2 mg/kg dissolved in a solution of 5% dimethyl sulfoxide (DMSO), 15% PEG-400, and 5% Tween-20) administered twice a day in a volume of 2 ml/kg, 3 days before starting a treatment with L-DOPA (Figure 1A). For the combined treatment, rapamycin was injected 1 h before the administration of L-DOPA plus benserazide. More experimental groups were analyzed to rule out: (1) possible effects due to the vehicle used to prepare rapamycin solution (6-OHDA, treated with vehicle + L-DOPA); (2) possible effects of rapamycin in parkinsonian animals (6-OHDA, treated with rapamycin); and (3) possible effects of rapamycin in control animals (sham-operated, treated with rapamycin; see Supplementary Methods section).

### Immunohistochemistry

After electrophysiological recordings, midbrain samples were postfixed overnight in a 4% paraformaldehyde solution and then cryoprotected through immersion in a 30% sucrose solution. Using a cryostat apparatus (Leica CM1850UV), brains were cut into coronal sections (30 μm of thickness) and stored sequentially in 0,1 M PB containing 0.02% sodium azide. We performed a DAB (3,3′-diaminobenzidine) immunohistological staining using the Ultratek HRP Anti-Polyvalent Staining System (ScyTek Laboratories), according to the manufacturer’s instructions. Briefly, sections were incubated with mouse anti-tyrosine hydroxylase (TH) primary antibody in PB-Triton X-100 0.3% for three overnight at 4°C (1:1,000; Merck Millipore MAB318). Finally, the sections were mounted on polysine slides (Thermo Fisher Scientific, Waltham, MA, USA) and then dehydrated and coverslipped with Eukitt (Sigma).

### Stereological Counts of Dopaminergic Neurons (TH^+^) of the SNc and Ventral Tegmental Area

To count TH^+^ cells in the hemispheres of 6-OHDA-lesioned rats, we performed a stereological analysis using the “Optical Fractionator” method of the Stereo Investigator System (MicroBrightField Europe; MBF Bioscience; Figure 1B). Briefly, we drew SNc and ventral tegmental area (VTA) regions using a 5× objective following the rat atlas coordinates (Paxinos and Watson, 2005). The count was carried out using a 40× objective, considering nine sections per animals (*n* = 3/4). Data were analyzed with a two-tailed Student’s *t*-test and are expressed as mean ± SEM.

### Abnormal Involuntary Movements Scoring

Abnormal involuntary movements, as an index of dyskinesia, were scored in L-DOPA-treated rats on alternate days (three times a week) for 2 weeks using an AIMs scale validated in parkinsonian rats (Cenci et al., 1998; Picconi et al., 2003; Cenci and Lundblad, 2007; Mellone et al., 2015; Ghiglieri et al., 2016). Each rat was placed into a transparent cage so that all movements could be observed from different angles. The AIMs rating session started 20 min after the first animal was injected with L-DOPA. Each rat was observed and rated for 1 min, every 20 min. The scoring session lasted up to 160 min. All animals were given a score (from 0 to 4) for each of the AIMs subtypes (limb, axial, orolingual; Cenci and Lundblad, 2007; Mellone et al., 2015). The total AIM score for each session was obtained by summing the score collected for each time point (Gardoni et al., 2006; Ghiglieri et al., 2016).

### Electrophysiological Experiments

Animals were killed by cervical dislocation 20 min after the last injection of L-DOPA to obtain corticostriatal slices for electrophysiological recordings. The corticostriatal slices from rat brains were cut (thickness, 240–280 μm) using a vibratome (Bagetta et al., 2012), transferred individually to a recording chamber, and submerged in a continuously flowing Krebs solution (room temperature; rate 2.5–3 ml/min) bubbled with a 95% O_2_–5% CO_2_ gas mixture. The composition of the solution was as follows, in mM: 126 NaCl, 2.5 KCl, 1.2 MgCl_2_, 1.2 NaH_2_PO_4_, 2.4 CaCl_2_, 10 D(+)-glucose, and 25 NaHCO_3_. For whole-cell patch, clamp recordings of SPNs (voltage clamped at −80 mV) were performed with borosilicate glass pipettes (4–6 MΩ) filled with the internal solutions composed in mM by 120 CsMeSO_3_, 10 CsCl, 8 NaCl, 2 MgCl_2_, 10 HEPES, 0.2 EGTA, 5 QX-314 chloride, 2 MgATP, 0.3 Na_3_GTP, and 10 TEA, adjusted to pH 7.3 with CsOH for synaptic measures, or 125 K + -gluconate, 10 NaCl, 1 CaCl_2_, 2 MgCl_2_, 10 HEPES, 2 MgATP, and 0.3 Na3GTP, adjusted to pH 7.3 with KOH for I/V calculations. Signals were amplified with a multiclamp 700B amplifier, recorded, and stored on PC using pClamp 9 (Molecular Devices, San Jose, CA, USA). Whole-cell access resistance was 10–30 MΩ. The recording electrodes were placed within the dorsolateral striatum. All the experiments were conducted in the continuous presence of Picrotoxin (50 μM), added to the perfusing solution, to block contamination of the corticostriatal excitatory postsynaptic potentials (EPSPs) with depolarizing GABA_A_-mediated potentials. Input resistances and injected currents were monitored throughout the experiments, and variations of these parameters higher than 20% lead to the rejection of the experiment. For intracellular recordings, sharp electrodes were filled with 2 M KCl (30–60 MΩ). Signals were recorded using an Axoclamp 2B amplifier (Molecular Devices), displayed on a separate oscilloscope, stored, and analyzed on a digital system (pClamp 9; Molecular Devices). Offline analysis was performed using Clampfit (Molecular Devices) and GraphPad Prism software. Glutamatergic EPSPs were evoked by electrical stimulation every 10 s, and to induce LTP, we used a high-frequency stimulation (HFS) protocol, consisting of three trains of 100 Hz, 3 s of duration, and 20 s of intertrain interval. During tetanic stimulation, the intensity was increased to suprathreshold levels. For the LTP protocol, at the beginning of intracellular recordings, magnesium ions were omitted from the medium to increase the NMDA-mediated component of EPSP (Bagetta et al., 2012; Ghiglieri et al., 2016). This protocol is needed to induce corticostriatal LTP in SPNs. To depotentiate a previously induced LTP, a LFS protocol of 10 min at a frequency of 2 Hz was delivered. Quantitative data on EPSP modifications induced by HFS protocol are expressed as a percentage of control, the latter representing the mean of responses recorded during a stable period (15–20 min) before the HFS. Current–voltage relationships were obtained by applying steps of current of 100 pA in both hyperpolarizing and depolarizing direction (from −400 to +200 pA) in order to measure the membrane ability to accommodate and fire in response to hyperpolarizing and depolarizing current steps.

Paired Student’s *t*-test was used to compare the pre-HFS (as mean of values at the −2nd and −4th min) vs. post-HFS (as mean of values at the 8th and 10th min) protocol and post-HFS (as mean of values at the 8th and 10th min) vs. post-LFS (as mean of the values at the 32nd and 34th min) protocol in the same cell population recorded from the same experimental group (paired). Unpaired Student’s *t*-test was used to compare post-LFS (as mean of the values at the 32nd and 34th min) values of the two experimental groups. The analyses were done using Prism 8.0 software (GraphPad Software).

### Biochemical Experiments

Contralateral and ipsilateral striatal tissues from sham-operated and 6-OHDA-lesioned rats treated with rapamycin or vehicle, and 6-OHDA-lesioned rats treated with rapamycin plus L-DOPA, or vehicle plus L-DOPA, were sonicated in 1% sodium dodecyl sulfate (SDS) and boiled for 10 min. Aliquots (2 μl) of the homogenate were used for protein determination using a Bio-Rad Protein Assay kit. Equal amounts of total proteins (15–30 μg) for each sample were loaded on precast 4% to 20% gradient gels (BioRad Laboratories, Hercules, CA, USA). Proteins were separated by SDS–polyacrylamide gel electrophoresis and transferred to PVDF membranes (GE Healthcare, Chicago, IL, USA) *via* the Trans Blot Turbo System (BioRad Laboratories). In order to investigate D1R/cAMP/PKA and mTOR signaling, the blots were incubated with phospho-GluA1 (Ser845; 1:500, p1160–845; PhosphoSolution, Aurora, CO, USA), phospho-p44/42 MAPK (ERK1/2; Thr202/Tyr204; 1:2,000; 9101; Cell Signaling Technology, Netherlands), and phospho-S6 ribosomal protein (Ser240/244; 1:1,000; 2215, Cell Signaling Technology, Danvers, MA, USA). The membranes were treated with stripping solution (StripAblot; EuroClone S.p.A, Milan, Italy), to remove primary antibodies according to the manufacturer’s protocol. After, antibodies against GluA1 (1:500; AB1504 (Merck Sigma, Germany), p44/42 MAPK (ERK1/2; 1:2,000; 9102; Cell Signaling Technology, Danvers, MA, USA), and S6 ribosomal protein (1:1,000; 2217; Cell Signaling Technology, Danvers, MA, USA) were used on the stripped membranes to estimate the total amount of respective proteins. The blots were also incubated with antibodies against GluA2/3 (1:500, 07598; Merck Sigma), GluN1 (1:1,000; 5704; Cell Signaling Technology, Danvers, MA, USA), GluN2A (1:1,000; G9038; Merck Sigma), and GluN2B (1:500, 4212; Cell Signaling Technology, Danvers, MA, USA). An antibody against TH (1:2,000, MAB318; Merck Sigma) was used to assess the severity of 6-OHDA lesions. DARPP-32 (1:500; 2306; Cell Signaling Technology, Danvers, MA, USA) was used to normalize protein levels of the analyzed striatal markers. The levels of phosphorylated proteins were normalized against the corresponding total proteins content normalized to DARPP-32 (Santini et al., 2007). All blots were incubated in horseradish peroxidase-conjugated secondary antibodies, and target proteins visualized by ECL detection (Pierce, Rockford, IL, USA), followed by quantification through the “Quantity One” software (BioRad Laboratories). Normalized values were then averaged and used for statistical comparisons. Statistical significance was determined by Two-way ANOVA followed by Bonferroni *post hoc* comparison. All representative blots shown in the figures arise from cut-out and pasted bands for reassembling the image. Of note, for each graph, the representative bands came from the same films.

## Results

### Chronic Rapamycin Does Not Affect the Extent of DA Denervation in 6-OHDA-Lesioned Rats

Adult male Wistar rats (*n* = 52) were used and lesioned through a unilaterally stereotaxic injection of the neurotoxin 6-OHDA in the ascending nigrostriatal pathway (MFB; Picconi et al., 2003; Paillé et al., 2010; Ghiglieri et al., 2016). The quantification of the remaining dopaminergic cells in the SNc was carried out to prove the 6-OHDA lesion degree through the stereological count of the TH enzyme-positive cells in coronal slices (L-DOPA alone vs. rapamycin + L-DOPA-treated rats; Figure 1B). The 6-OHDA lesions in the MFB region of rat brains led to a complete dopaminergic cell loss in the regions of SNc (>90%) and greater than 70% of TH+ cell loss in VTA (Figure 1C, left panel) of both treated groups (L-DOPA alone vs. rapamycin + L-DOPA; Figure 1C, right panel).

### Rapamycin Treatment Reduces Abnormal Involuntary Movements

**Figure 2:**
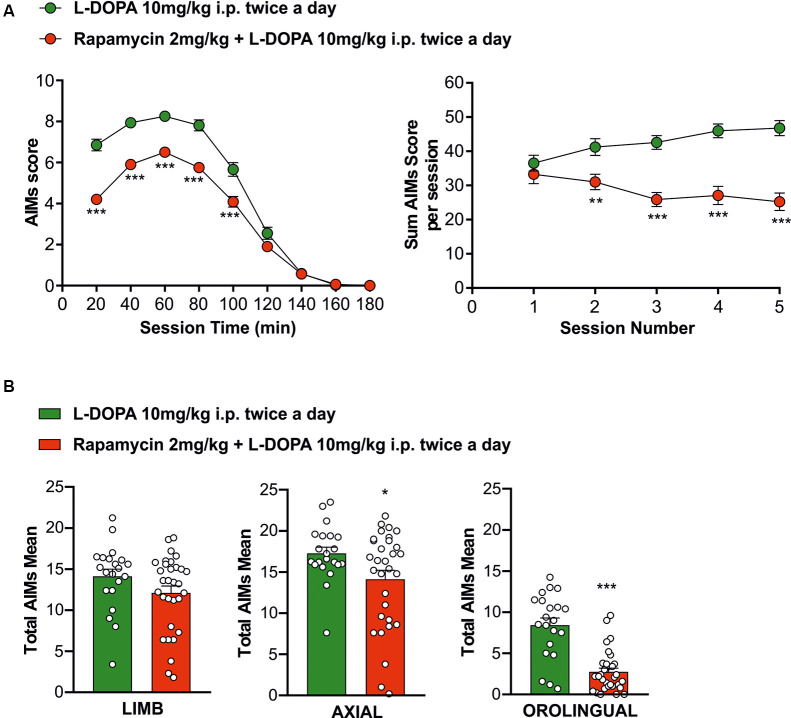
Behavioral testing to evaluate L-DOPA treatment effects in the experimental groups. (**A**, left panel) The rapamycin + L-DOPA-treated group (*n* = 21 rats) showed a significantly less occurrence of dyskinetic behaviors compared to L-DOPA group (*n* = 21 rats), during the whole treatment period from the 20th minute to the 100th minute (***p < 0.001). (**A**, right panel) L-DOPA-treated rats showed a compromising trend of the dyskinesia compared to rapamycin + L-DOPA-treated rats (**p < 0.01; ***p < 0.001). **(B)** Behavioral antidyskinetic effect of rapamycin treatment mainly concerning axial (*p < 0.05) and orolingual (***p < 0.001).

It is known that, similarly to PD patients, chronic treatment with L-DOPA causes the onset of LID in an experimental rodent model of PD obtained with the injection of the 6-OHDA neurotoxin in the MFB (Picconi et al., 2003, 2011; Cenci and Lundblad, 2007; Lindgren et al., 2010; Ghiglieri et al., 2016). To study the effect of mTORC1 inhibitor rapamycin, one group of 6-OHDA rats (*n* = 31) has been chronically treated with L-DOPA alone (10 mg/kg plus 6 mg/kg benserazide, twice a day, 10 AM and 5 PM, i.p.) for 2 weeks, whereas the second group of animals (*n* = 21) has been pretreated with rapamycin (2 mg/kg, twice a day, 10 AM and 5 PM, i.p.) for 3 days and later in combination with L-DOPA twice a day (10 AM and 5 PM), for 2 weeks. Behavioral analysis of AIMs indicate that L-DOPA caused the development of dyskinetic movements in both experimental groups, however, on a different extent of severity [Figure 2A, right panel; unpaired Student’s *t*-test L-DOPA alone vs. rapamycin + L-DOPA: Session 1 *t* = 0.8300, *df* = 49, not statistically significant (n.s.); Session 2 *t* = 2.966, *df* = 48, **p < 0.01; Session 3 *t* = 5.219, *df* = 44, ***p < 0.001; Session 4 *t* = 5.203, *df* = 47, ***p < 0.001; Session 5 *t* = 5.346, *df* = 42, ***p < 0.001]. Consistently, the scores obtained in all the AIMs test sessions showed a significant difference between the two experimental groups at 20, 40, 60, 80, and 100 min (Figure 2A, left panel, L-DOPA alone vs. rapamycin + L-DOPA, green circles and red circles, respectively; two-way ANOVA interaction factor, session × treatment interaction, *F*_(8,2024)_ = 15.79, Bonferroni *post hoc*, ***p < 0.001). The reduction of dyskinesia intensity (Figure 2B) concerned axial (unpaired Student’s *t*-test *t* = 2.190, *df* = 50, *p < 0.05) and, most prominently, orolingual components (unpaired Student’s *t*-test *t* = 6.331, *df* = 50, ***p < 0.001) compared to the values observed in animals treated with L-DOPA alone. To rule out any possible effect due to the vehicle, used to dissolve rapamycin, we treated a group of rats with the vehicle alone followed, 1 h later, by L-DOPA, and we scored the AIMs of these groups. Abnormal involuntary movements were found similar over time as expected, suggesting the absence of effects of the vehicle itself (Supplementary Results and Supplementary Figure S1). Overall, the cotreatment with rapamycin was able to limit the detrimental effects of chronic L-DOPA administration by significantly reducing dyskinetic responses. Interestingly, this effect is observed in the intensity of the AIMs and mainly in the orolingual component.

### Rapamycin Does Not Affect Intrinsic Properties of Striatal Projection Neurons

**Figure 3:**
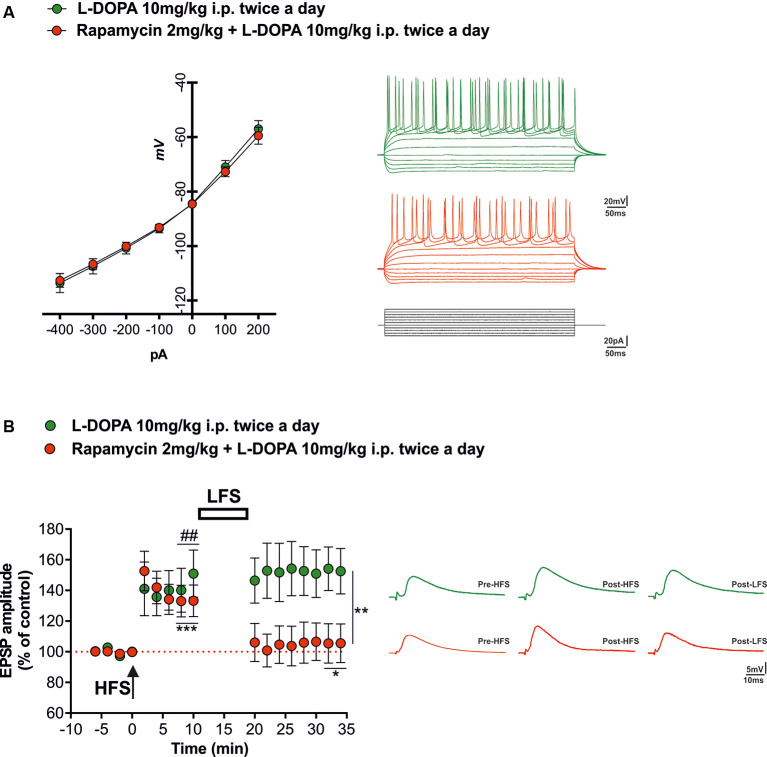
Synaptic plasticity in spiny projection neurons (SPNs) of L-DOPA vs. rapamycin + L-DOPA-treated rats. (**A**, left panel) Current-voltage curves obtained during current-clamp recordings of SPNs for each experimental group. (**A**, right panel) Tonic firings of SPNs obtained through intracellular and whole-cell patch clamp recordings of corticostriatal slices from animals treated with L-DOPA (green line) and with a combination of rapamycin plus L-DOPA (red line), in response to hyperpolarizing and depolarizing current pulses. (**B**, left panel) Time course of the high-frequency stimulation (HFS)-induced long-term potentiation (LTP) and low-frequency stimulation (LFS)-induced depotentiation in corticostriatal SPNs. (**B**, right panel) Representative corticostriatal excitatory postsynaptic potential (EPSP) amplitude traces showing the recovery of the LTP in each experimental group (L-DOPA and rapamycin + L-DOPA). The application of a HFS protocol induced LTP in both groups (L-DOPA ##*p* < 0.01; rapamycin + L-DOPA ***p < 0.001). LFS induced depotentiation only in the group treated with both rapamycin and L-DOPA (*p < 0.05 compared to 8-10 min post HFS; **p < 0.01 compared to L-DOPA alone group).

Electrophysiological analyses carried out through whole-cell patch clamp and intracellular recordings of dorsolateral SPNs in rats of the two experimental groups (L-DOPA alone vs. rapamycin + L-DOPA) showed similar resting membrane potentials (RPMs) and firing patterns (Figure 3A, right panel). The mean RPM was −84 mV (±3 mV) in the SPNs of L-DOPA alone group and −85 mV (±5 mV) in those of the rapamycin + L-DOPA group. No differences were observed between the experimental groups in the responses of the SPNs to hyperpolarizing and depolarizing current pulses (Figure 3A, left panel; two-way ANOVA, time effect (*F*_(6,138)_ = 137.4; Bonferroni *post hoc* test, n.s.), treatment effect (*F*_(1,23)_ = 0.01517; Bonferroni *post hoc* test, n.s.), and time × treatment interaction (*F*_(6,138)_ = 0.1667; Bonferroni *post hoc* test, n.s.).

### Pharmacological Inhibition of mTORC1 Activity Prevents the Loss of Striatal Synaptic Depotentiation

Electrophysiological recordings show that corticostriatal LTP, in agreement with previous reports (Picconi et al., 2003, 2008; Cerovic et al., 2015; Ghiglieri et al., 2016), was restored in all the SPNs recorded from 6-OHDA-lesioned rats chronically treated with L-DOPA alone (Figure 3A, green circles; paired Student’s *t*-test, pre-HFS vs. 10 min post-HFS *t* = 4.187 *df* = 12, ^##^*p* < 0.01, *n* = 6 cells). Rapamycin, when coadministered, preserved the effects of L-DOPA on the ability of SPNs to express LTP (Figure 3B, red circles; paired Student’s *t*-test pre-HFS vs. 10 min post-HFS, *t* = 4.763, *df* = 21, ***p < 0.001, *n* = 13 cells). We then tested if the combined treatment was able to prevent the loss of bidirectional plasticity, which can be observed in control conditions through the application of a LFS protocol in SPNs that expressed LTP. As hypothesized, only SPNs of animals treated with the combined administration of rapamycin plus L-DOPA were able to respond with synaptic depotentiation of their EPSPs amplitude (Figure 3B, red circles, paired Student’s *t*-test post-HFS vs. 14 min post-LFS, *t* = 2.349, *df* = 21, *p < 0.05, *n* = 13 cells), whereas the SPNs of the L-DOPA alone group were unable to reverse synaptic potentiation (Figure 3B, green circles, paired Student’s *t*-test post-HFS vs. 14 min post-LFS, *t* = 1.390, *df* = 11, n.s., *n* = 6 cells; unpaired Student’s *t*-test L-DOPA post-LFS vs. rapamycin plus L-DOPA post-LFS, *t* = 3.278, *df* = 36, **p < 0.01).

### Effect of Rapamycin Upon Striatal D1R-Dependent Signaling in L-DOPA-Treated 6-OHDA-Lesioned Rats

Based on the established role of mTORC1 pathway in modulating LID onset and severity through an aberrant activation of the striatal DA D1R-dependent signaling (Santini et al., 2007, 2009; Decressac and Björklund, 2013; Brugnoli et al., 2016), we sought to investigate the effect of mTORC1 inhibition by rapamycin upon PKA/ERK and mTOR activity, in the unilaterally 6-OHDA-lesioned rats, treated with L-DOPA.

First, in 6-OHDA-lesioned rats, treated with L-DOPA, with or without rapamycin, we evaluated the phosphorylation state of GluA1 (pGluA1) at Ser845 residue, which represents one of the main striatal cAMP-dependent protein kinase (PKA) target site (Roche et al., 1996; Snyder et al., 2000; Håkansson et al., 2006).

**Figure 4:**
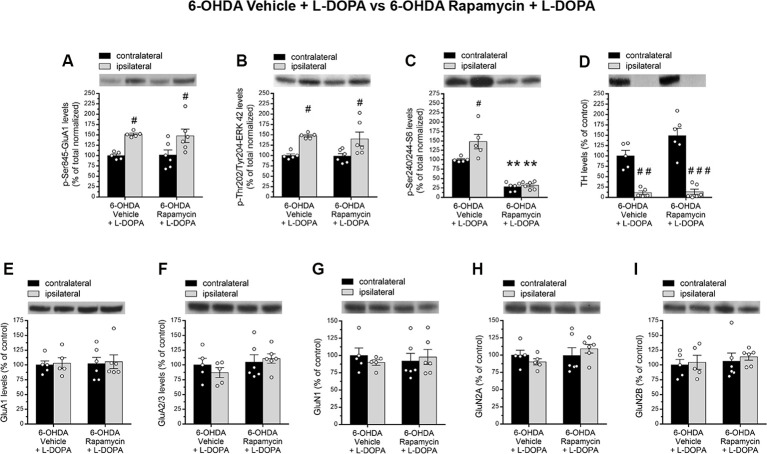
**(A–I)** Western blot analysis of indicated proteins from contralateral and ipsilateral striata of 6-OHDA-lesioned rats treated with vehicle + L-DOPA (*n* = 5) or rapamycin + L-DOPA (*n* = 6). **(A–C)** Phosphorylation levels of GluA1 at Ser845, ERK42 at Thr202/Tyr204, and S6 at Ser240/244. **(D)** Measurement of TH levels in contralateral and ipsilateral striata of lesioned rats. **(E,F)** Striatal GluA1 and GluA2/3 glutamate AMPA receptor subunits and **(G–I)** GluN1, GluN2A, and GluN2B N-methyl-D-aspartate (NMDA) receptor subunit levels. All data are expressed as mean ± SEM. ^#^*p* < 0.05, ^##^*p* < 0.01 compared to contralateral striatum of 6-OHDA vehicle + L-DOPA-treated rats; ^#^*p* < 0.05, ^###^*p* < 0.0001 compared to contralateral striatum of 6-OHDA rapamycin + L-DOPA-treated rats; **p < 0.01 compared to contralateral striatum of 6-OHDA vehicle + L-DOPA-treated rats [two-way analysis of variance (ANOVA), followed by Bonferroni multiple-comparisons test].

Here, we showed that, regardless rapamycin coadministration, L-DOPA produced an increase of striatal pGluA1 levels in all 6-OHDA-lesioned rats. Indeed, two-way ANOVA showed a significant lesion effect (*F*_(1,18)_ = 18.02, *p* = 0.0005), a nonsignificant (n.s.) rapamycin treatment effect (*F*_(1,18)_ = 0.01475, *p* = 0.9047; Figure 4A), and n.s. lesion × treatment interaction on pGluA1 levels (*F*_(1,18)_ = 0.03839, *p* = 0.8469; Figure 4A). Moreover, L-DOPA administration triggered greater striatal pERK42 levels also when coinjected with rapamycin to 6-OHDA-lesioned rats. Indeed, two-way ANOVA showed a significant lesion effect (*F*_(1,18)_ = 19.32, *p* = 0.0003), a n.s. rapamycin treatment effect (*F*_(1,18)_ = 0.2099, *p* = 0.6523), and n.s. lesion × treatment interaction on pERK42 levels (*F*_(1,18)_ = 0.088, *p* = 0.7701; Figure 4B).

Then, in order to confirm the ability of rapamycin treatment to inhibit mTOR signaling in rats, we analyzed the effect of this drug on the striatal levels of phospho-Ser240/244-S6 (pS6). As expected, in 6-OHDA-lesioned striatum, L-DOPA alone promoted mTORC1 activation (Santini et al., 2009; Decressac and Björklund, 2013; Eshraghi et al., 2020), as revealed by higher pS6 (Santini et al., 2009; Figure 4C). Conversely, rapamycin administration induced a significant reduction of pS6 levels in both ipsilateral and contralateral side of lesioned rats cotreated with L-DOPA. According to this, two-way ANOVA revealed a significant lesion effect (*F*_(1,18)_ = 7.699, *p* = 0.0123), a significant rapamycin treatment effect (*F*_(1,18)_ = 101, *p* < 0.0001), and a significant lesion × treatment interaction on striatal pS6 levels (*F*_(1,18)_ = 5.649, *p* = 0.0288; Figure 4C). The extent of DA denervation was assessed by Western blot analysis in both experimental groups. Data revealed a dramatic decrease (average >90%) of the striatal TH immunolabeling in the ipsilateral side to the lesion, when compared to the contralateral side (two-way ANOVA indicated a significant lesion effect: *F*_(1,18)_ = 83.34, *p* < 0.0001; Figure 4D).

Alongside a crucial role of glutamatergic neurotransmission in driving, among others, PD-derived motor complications, including LID (Gardoni et al., 2006; Calabresi et al., 2008; Sgambato-Faure and Cenci, 2012), we investigated the influence of rapamycin treatment upon AMPA and NMDA receptor subunits expression in total striatal homogenates.

In line with previous findings (Gardoni et al., 2006), neither 6-OHDA vehicle + L-DOPA nor 6-OHDA rapamycin + L-DOPA groups affected the total expression levels of GluA1 and GluA2/3 (AMPA receptors), GluN1, GluN2A, and GluN2B (NMDA receptors). Indeed, two-way ANOVA, on GluA1, showed a n.s. lesion effect: *F*_(1,18)_ = 0.1038, *p* = 0.7510; n.s. treatment effect: *F*_(1,18)_ = 0.04373, *p* = 0.8367; on GluA2/3, showed a n.s. lesion effect: *F*_(1,18)_ = 0.1228, *p* = 0.7301; and a n.s. treatment effect: *F*_(1,18)_ = 1.976, *p* = 0.1769; on GluN1, showed a n.s. lesion effect: *F*_(1,18)_ = 0.04441, *p* = 0.8355; and a n.s. treatment effect: *F*_(1,18)_ = 0.0001, *p* = 0.9968; on GluN2A, a n.s. lesion effect: *F*_(1,18)_ = 0.0006, *p* = 0.9981; and a n.s. treatment effect: *F*_(1,18)_ = 1.337, *p* = 0.2627; on GluN2B, a n.s. lesion effect: *F*_(1,18)_ = 0.3067, *p* = 0.5866; and a n.s. treatment effect: *F*_(1,18)_ = 0.5301, *p* = 0.4759; Figures 4E–I).

### Effect of Rapamycin Upon Striatal D1R-Dependent Signaling in 6-OHDA-Lesioned and Sham-Operated Rats

**Figure 5:**
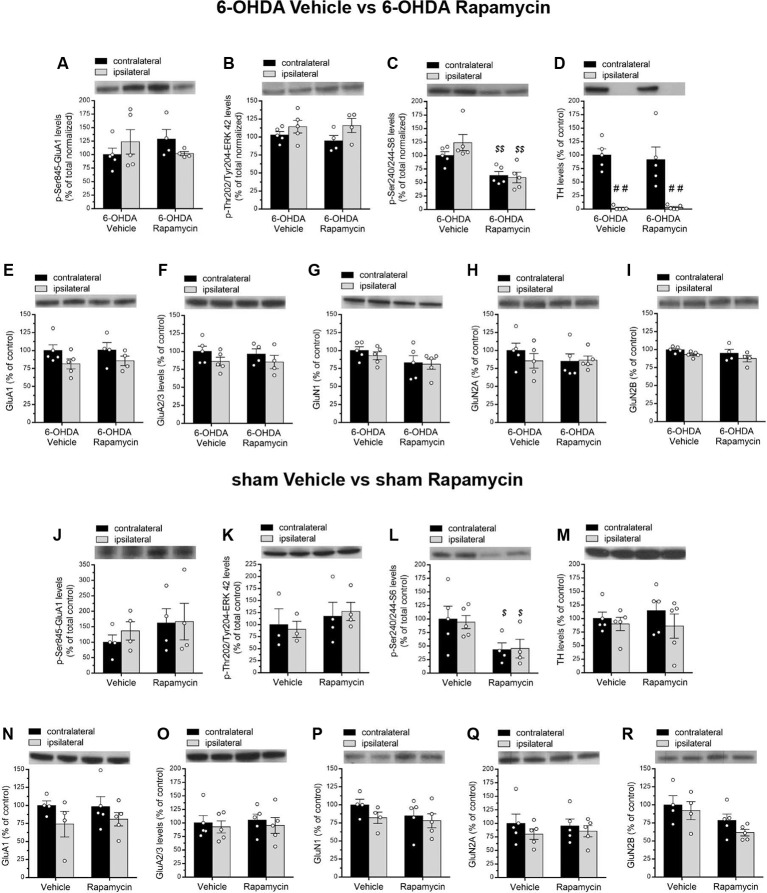
**(A–I)** Western blot analysis of indicated proteins from contralateral and ipsilateral striata of 6-OHDA-lesioned rats treated with vehicle or rapamycin. **(A,B)** p-Ser845-GluA1 and p-Thr202/Tyr204-ERK42 protein levels in dopamine (DA) denerveted rats treated with vehicle (*n* = 5) or rapamycin (*n* = 4). **(C,D)** p-Ser240/244-S6 and TH protein levels (*n* = 5/treatment). **(E,F)** Protein expression of the subunits GluA1 and GluA2/3 of AMPA-type glutamate receptors in vehicle (*n* = 5) and rapamycin (*n* = 4) treated rats lesioned with 6-OHDA. **(G,H)** GluN1 and GluN2A NMDA receptor subunit levels in 6-OHDA vehicle or 6-OHDA rapamycin-treated rats (*n* = 5/treatment). **(I)** Expression of the GluN2B subunit in vehicle (*n* = 5) or rapamycin (*n* = 4) treated rats lesioned with 6-OHDA. **(J–R)** Western blot analysis of indicated proteins from contralateral and ipsilateral striata of sham-operated rats treated with vehicle or rapamycin (*n* = from 3 to 5/treatment). **(J–L)** Phosphorylation levels of GluA1 at Ser845, ERK42 at Thr202/Tyr204, and S6 at Ser240/244. **(M)** Tyrosine hydroxylase protein levels in contralateral and ipsilateral striata from sham-operated group. Protein expression of **(N,O)** AMPA (GluA1 and GluA2/3) and **(P–R)** NMDA glutamate receptor subunits (GluN1, GluN2A, and GluN2B). All data are expressed as mean ± SEM. Statistical significance was determined by two-way ANOVA, followed by Bonferroni multiple-comparisons test, ^##^*p* < 0.01 for TH protein levels. For phospho-S6 levels (panels **C,L**), two-way ANOVA returned a significant treatment effect (^$$^*p* < 0.001 and ^$^*p* < 0.05 for panels **C,L**, respectively).

Without L-DOPA administration, our biochemical results showed no main effect of 6-OHDA lesion and rapamycin treatment on pGluA1 and pERK42 levels (two-way ANOVA; pGluA1, lesion effect: *F*_(1,14)_ = 0.009265, *p* = 0.9247; treatment effect: *F*_(1,14)_ = 0.04814, *p* = 0.8295; pERK42, lesion effect: *F*_(1,14)_ = 4.392, *p* = 0.0548; treatment effect: *F*_(1,14)_ = 0.1883, *p* = 0.6709; Figures 5A,B).

On the other hand, rapamycin treatment significantly reduced pS6 levels, regardless of the lesion side (two-way ANOVA, lesion effect: *F*_(1,16)_ = 0.9604, *p* = 0.3417; treatment effect: *F*_(1,16)_ = 24.06, *p* = 0.0002; Figure 5C). The evaluation of TH levels revealed an almost total DA depletion in the lesioned side of both vehicle and rapamycin groups (two-way ANOVA, lesion effect: *F*_(1,16)_ = 50.78, *p* < 0.0001, Figure 5D). Moreover, rapamycin did not affect AMPA and NMDA receptor subunits in total striatal homogenates, thus showing protein levels comparable to those found in 6-OHDA-lesioned vehicle group (two-way ANOVA, lesion × treatment interaction; GluA1: *F*_(1,14)_ = 0.04055, *p* = 0.8433; GluA2/3: *F*_(1,14)_ = 0.03784, *p* = 0.8486; GluN1: *F*_(1,16)_ = 0.1268, *p* = 0.7264; GluN2A: *F*_(1,16)_ = 0.7266, *p* = 0.4066; GluN2B: *F*_(1,14)_ = 0.008487, *p* = 0.9279; Figures 5E–I).

Finally, we documented that rapamycin did not produce any significant alterations of all analyzed targets in sham-operated rats, when compared to vehicle group. Consistently, two-way ANOVA indicated a n.s. sham-lesion × treatment interaction: pGluA1: *F*_(1,12)_ = 0.1423, *p* = 0.7126; pERK42: *F*_(1, 10)_ = 0.1521, *p* = 0.7047; GluA1: *F*_(1,14)_ = 0.1061, *p* = 0.7494; GluA2/3: *F*_(1,16)_ = 0.009488, *p* = 0.9236; GluN1: *F*_(1,14)_ = 0.32, *p* = 0.5806; GluN2A: *F*_(1,16)_ = 0.1624, *p* = 0.6923; GluN2B: *F*_(1,14)_ = 0.2181, *p* = 0.6477; Figures 5J,K,N–R). Again, rapamycin injection caused an overall significant reduction in both ipsilateral and contralateral sides of pS6 levels in sham-operated rats (two-way ANOVA, treatment effect: *F*_(1,14)_ = 8.875, *p* = 0.0100; Figure 5L).

## Discussion

For the first time, here we demonstrated that abnormal striatal mTORC1 pathway activation by chronic L-DOPA is a causal factor to the loss of synaptic depotentiation in SPNs of parkinsonian rats. Consistent with this assumption, pharmacological inhibition of mTOR preserved the ability of SPNs to express LTP, which can be depotentiated, preventing, *de facto*, the loss of bidirectional synaptic plasticity. In addition, we documented that inhibition of mTORC1 pathway by pretreatment with rapamycin and its subsequent coadministration with L-DOPA improved the therapeutic effect of this anti-PD drug by limiting the emergence of specific AIMs (axial and orolingual).

In the last two decades, it has been demonstrated that changes in striatal function associated to L-DOPA-induced side effects occur in neurons of the direct striatonigral pathway (D1Rs) through hyperactivation of the canonical cAMP/PKA/DARPP-32 cascade (Calabresi et al., 2000b; Svenningsson et al., 2000; Picconi et al., 2011) and modification of signaling pathway that includes ERK and its ability to promote the activation of mTORC1 signaling under DA depletion (Valjent et al., 2005; O’Sullivan et al., 2008; Subramaniam et al., 2011; Santini et al., 2012).

In rodent models of LID, a significant increase in striatal mTORC1 pathway activation (Santini et al., 2009, 2012) is determined by maladaptive processes caused by the abnormal activation of ERK (Gerfen et al., 2002; Cerovic et al., 2015). Consistent with this, enhanced phosphorylation state of mTORC1 targets such as p70 ribosomal S6 kinases (S6K), ribosomal protein S6 (rpS6), and initiation factor 4E-binding protein (Gingras et al., 2001; Ruvinsky and Meyuhas, 2006) have been reported in the striatum of dyskinetic mice and rats (Santini et al., 2009; Subramaniam et al., 2011; Decressac and Björklund, 2013). Also dysfunctional increased mTORC1 signaling in the striatum of dyskinetic animals occurs selectively in SPNs expressing hypersensitized DA D1Rs (Santini et al., 2009) as demonstrated by the observation of the effects of DA D1R antagonist SCH23390, which abolishes the phosphorylation of S6Ks induced by L-DOPA, whereas the inhibition of D2Rs with raclopride exerts no effects (Santini et al., 2009). It has hence been postulated that a hypersensitive DA D1R transmission plays a crucial role in striatal microcircuit homeostasis, as an intact dopaminergic neurotransmission is the main modulatory system that controls the responsiveness of SPNs to glutamatergic inputs coming from the cortex ad the thalamus (Surmeier et al., 2014).

Relevant to corticostriatal synaptic plasticity, a control of glutamatergic activity and a correct activation of D1Rs are specifically needed for the induction of striatal LTP (Shen et al., 2008).

Chronic L-DOPA administration allows, on the one hand, to restore LTP of SPNs, although, on the other hand, in animals that express LID, it correlates to the absence of synaptic depotentiation, inducible in physiological conditions by a subsequent application of a LFS protocol (Picconi et al., 2003). The loss of this homeostatic plasticity is coupled with the aforementioned hyperactivation of the D1Rs, resulting in an increase of D1/PKA/DARPP-32 signaling pathway (Picconi et al., 2003), which determines synaptic saturation and reinforcement of nonphysiological motor circuits within the basal ganglia network.

Based on these findings, in our study, we evaluated the role of mTORC1 signaling in modulating corticostriatal plasticity, LID, and biochemical features induced by chronic L-DOPA in 6-OHDA rat model. It is known that initial events that make possible LTP induction also potently activate mTORC1 activity that contributes to long-term plasticity (Cammalleri et al., 2003; Tsokas et al., 2007). This evidence is not a distinct feature of striatum as also late phase of LTP in rodent hippocampal slices is blocked by rapamycin-induced mTORC1 inhibition (Tang et al., 2002; Cammalleri et al., 2003), an observation that confirms the conserved role of mTORC1 in protein synthesis–dependent long-lasting synaptic potentiation.

**Figure 6:**
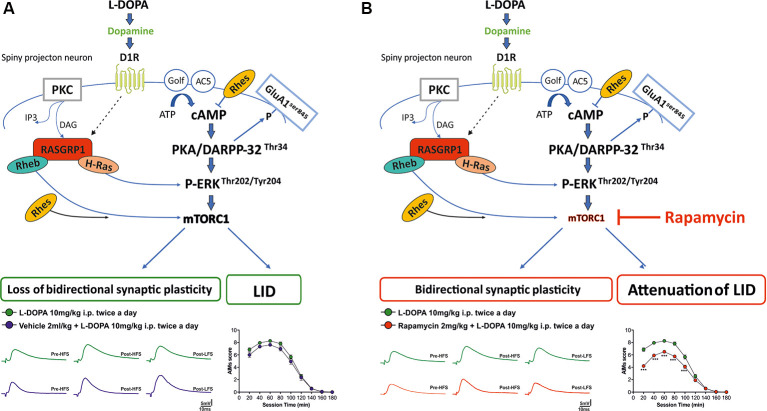
Schematic diagram illustrating **(A)** striatal D1/cAMP/PKA, pERK, and mTOR signaling cascade in a rat model of L-DOPA-induced dyskinesia; **(B)** the selective effect of rapamycin on mTORC1 signaling in a rat model of L-DOPA-induced dyskinesia.

In our experiments, we confirmed that chronic L-DOPA in lesioned rats is able to elicit LID and striatal bidirectional synaptic plasticity alterations, and in turn, these effects were linked to overactivation of striatal D1/cAMP/PKA, pERK1/2 and mTOR signaling pathways. Remarkably, here we reported that rapamycin preadministration in L-DOPA-treated 6-OHDA rats, by reducing S6 phosphorylation levels, fully prevented the loss of synaptic depotentiation and in turn attenuated the severity of axial and orolingual dyskinesia. Interestingly, rapamycin preadministration in L-DOPA-treated lesioned rats failed to affect D1/PKA/pGluA1^ser 845^ and pERK42 signaling, known to act upstream to mTOR pathway activation (Figure 6).

In support of our previous findings (Picconi et al., 2003; Calabresi et al., 2016), the current data strengthen the existence of a functional relationship between coordinated voluntary movement and striatal synaptic plasticity. Consistent with this idea, we confirmed that rapamycin administration by blocking striatal bidirectional plasticity alterations was able to significantly attenuate the severity of specific subtypes of AIMs in dyskinetic rats.

Remarkable, in contrast with previous studies in mouse models, showing an almost complete block of AIMs, here we report a distinctive profile in the behavioral effect of rapamycin. This discrepancy might be due to the different regimen of L-DOPA used or to interspecific differences that may account for species-specific engagement of motor components into behavioral responses to L-DOPA. Another study, carried out in rats, showed a more remarkable reduction of AIMs compared to the one shown here (Decressac and Björklund, 2013). The authors used a rapamycin derivative (temsirolimus, an ester of rapamycin) and a different drug dosage regimen, differences that may account for the milder effects seen here. In our study, we also tested the biochemical and electrophysiological effects of rapamycin in lesioned and intact rat striatum, demonstrating that mTORC1 inhibition, *per se*, did not affect NMDA and AMPA subunit composition and failed to perturb induction and maintenance of activity-dependent plasticity in SPNs of 6-OHDA-lesioned (Figure 5 and Supplementary Figure S2). Moreover, no detrimental effects were observed in rapamycin-treated sham-operated rats (Figure 5), as previously demonstrated also with *in vitro* incubation of corticostriatal slices (Ghiglieri et al., 2010). Furthermore, rapamycin did not perturb the effects of L-DOPA on corticostriatal glutamatergic synapses resulting in the recovery of LTP, as LTP maintenance was not affected in SPNs of cotreated rats (Supplementary Figure S2).

Overall, our observations indicate that the inhibition of mTORC1 through chronic rapamycin administration may prevent the loss of downscaling of the striatal synaptic potentiation through a fine control of a selected step in the signaling cascade that brings to consolidate aberrant plastic changes. In addition, our molecular data indicate that, in a dyskinetic PD rat model, rapamycin administration prevented the activation of striatal mTORC1 signaling without influencing the overactivation of PKA/DARPP-32 and pERK pathways, as well as the total protein expression of AMPA and NMDA receptor subunits. A possible limitation of this study is that, despite the recognized involvement of hyperactivity of D1 receptor signaling in LID, no clear segregation has been observed in the responses of SPNs to the different drug treatments during the electrophysiological experiments, which brought to consistent results across experimental groups. This aspect deserves further investigation to provide additional insights into new mechanisms by which rapamycin can influence striatal plasticity either by acting on elements of the striatal microcircuit other than SPNs (Goldshmit et al., 2015) or by exerting immunomodulatory effects that have been found to be associated to antidyskinetic actions (Boi et al., 2019).

Furthermore, the molecular mechanisms by which a dysfunctional mTORC1 overactivation exerts *in vivo* and electrophysiological alterations in dyskinetic condition through abnormal changes in specific striatal protein expression are still obscure. Future studies will be aimed at identifying the specific striatal mTORC1-related targets ultimately responsible for the observed behavioral and synaptic changes found under LID.

In conclusion, the current study sheds new light on the molecular mechanisms of action of mTORC1 in regulating corticostriatal bidirectional plasticity. Our results can be useful in designing drug repurposing approaches and/or in the development of new combined treatments able to normalize LID and synaptic plasticity by concurrently interfering with multiple strategic targets in the signaling pathways downstream D1 and NMDA receptor activations.

## Data Availability Statement

The datasets generated for this study are available on request to the corresponding author.

## Ethics Statement

The animal study was reviewed and approved by the Institutional Animal Care and Use Committee (Organismo Per il Benessere Animale, OPBA) at IRCCS Fondazione Santa Lucia and by the Italian Ministry of Health (D.Lgs. 295/2012-A and D.Lgs. 26/2014; prot. 1296/2015 PR) according to The Guidelines of The European Union Council (2010/6106/UE).

## Author Contributions

VG designed the study supervised by AU and BP. AC, ADM, ADR, FC, GM, GN, MM, VC, and VG acquired data. AC, ADM, ADR, FC, GM, GN, VC, and VG processed and analyzed the data. VC wrote the first draft. FN and LA critically revised the manuscript. BP, AU, VG, PC, and FN revised the article for critical discussion and content.

## Conflict of Interest

The authors declare that the research was conducted in the absence of any commercial or financial relationships that could be construed as a potential conflict of interest.
