# Supplementary material for: Rapamycin, by Inhibiting mTORC1 Signaling, Prevents the Loss of Striatal Bidirectional Synaptic Plasticity in a Rat Model of L-DOPA-Induced Dyskinesia
Source: Front Aging Neurosci. 2020 Aug 11;12:230. doi: 10.3389/fnagi.2020.00230 (PMC7431470; doi:10.3389/fnagi.2020.00230)
Supplement: Supplementary file 1 [file Data_Sheet_1.pdf]

SUPPLEMENTARY DATA

METHODS

6-hydroxydopamine hydrochloride (6-OHDA) nigrostriatal injection and apomorphine test

For the unilateral nigrostriatal DA denervation, adult male Wistar rats (n=40) (Charles River Laboratories, Calco, Italia) were deeply anesthetized with Zoletil (50/50 mg/ml tiletamine and zolazepam) and Rompun (20 mg/ml xylazine hydrochloride) and were stereotaxically injected with 6-OHDA (3 µg/µl in NaCl solution containing 0.1% ascorbic acid, volume 4 µl) into the medial forebrain bundle (MFB) of the left hemisphere, at a rate of 0.38 µl/min, using a standard 10 µl Hamilton syringe (Ghiglieri et al., 2016). Coordinates were calculated in mm from the bregma and the dura mater surface: tooth bar -2.3, anteroposterior (AP) -4.4 mm; mediolateral (ML) +1.2 mm and dorsoventral (DV) -7.8 mm (Paxinos et al., 1985; Paille et al., 2010; Bagetta et al., 2012; Ghiglieri et al., 2016).

In order to assess the efficacy of the 6-OHDA lesion, fifteen days after the surgery, lesioned rats were tested with a subcutaneous injection of apomorphine (0.05 mg/kg; dissolved in in 0.9% NaCl saline) and turns contralateral to the lesioned side were counted for 40 minutes using an automatic rotometer (Ungerstedt, 1971; Picconi et al., 2008). Two months after the surgery 42 fully lesioned rats, showing more than 200 contralateral turns, were subjected to drug treatments, behavioral test, molecular and electrophysiological experiments. This time interval from surgery was to avoid any interference of apomorphine treatment with the DA-dependent plastic changes occurring after dopaminergic denervation (Herrera-Marschitz and Ungerstedt, 1984; Schwarting and Huston, 1996; Picconi et al., 2004).

Drug treatments

Experiments were conducted using three experimental groups. One group (n=21 rats) was treated for two weeks with only L-DOPA (10 mg/kg L-DOPA plus 6mg/kg benserazide, intraperitoneal - i.p. -

twice a day; dissolved in 0.9% NaCl saline). A second group (n=10 rats) was treated with Vehicle (2 ml/kg of 5% dimethyl sulfoxide (DMSO), 15% PEG-400, and 5% Tween-20, intraperitoneal - i.p.) administered twice a day, three days before starting a treatment with L-DOPA. For the combined treatment, Vehicle was injected one hour before the administration of L-DOPA. A third group (n=9 rats) was treated with Rapamycin (2 mg/kg dissolved in a solution of 5% dimethyl sulfoxide (DMSO), 15% PEG-400, and 5% Tween-20) administered twice a day in a volume of 2 ml/kg, three days before starting a treatment with L-DOPA. For the combined treatment, Rapamycin was injected one hour before the administration of L-DOPA plus benserazide.

Abnormal Involuntary Movements (AIMs) scoring

Abnormal involuntary movements (AIMs), as indexes of dyskinesia, were scored in L-DOPA-treated rats on alternate days (three times a week) for two weeks using an AIMs scale validated in parkinsonian rats (Cenci et al., 1998; Picconi et al., 2003; Cenci and Lundblad, 2007; Mellone et al., 2015; Ghiglieri et al., 2016). Each rat was placed into a plexiglass cage with transparent walls so that all movements could be observed from different angles. The AIMs rating session started 20 minutes after the injected with L-DOPA. Each rat was observed and rated for one minute, every 20 minutes. The scoring session lasted up to 160 minutes. All animals were given a score (from 0 to 4) for the rotational behavior and for each of the AIMs subtypes (limb, axial, orolingual) (Cenci and Lundblad, 2007; Mellone et al., 2015). The total AIM score for each session was obtained by summing the score collected for each time point (Cenci and Lundblad, 2007).

Electrophysiological experiments

Animals were killed by cervical dislocation 20 minutes after the last injection of L-DOPA to obtain corticostriatal slices for electrophysiological recordings. The corticostriatal slices (thickness, 240-280 μm) obtained from rat brains using a vibratome (Bagetta et al., 2012), were then transferred individually to a recording chamber and submerged in a continuously flowing Krebs' solution (room

temperature; flow rate 2.5–3 ml/min) continuously bubbled with a 95% O_2–5% CO_2 gas mixture. The composition of the solution was in mM: 126 NaCl, 2.5 KCl, 1.2 $MgCl_2$, 1.2 NaH_2PO_4, 2.4 $CaCl_2$, 10 D(+)-Glucose and 25 $NaHCO_3$. For whole-cell patch clamp, recordings of SPNs (voltage clamped at -80 mV) were performed with borosilicate glass pipettes (4-6 MΩ) filled with the internal solutions composed in mM by 120 $CsMeSO_3$, 10 CsCl, 8 NaCl, 2 $MgCl_2$, 10 HEPES, 0.2 EGTA, 5 QX-314 chloride, 2 MgATP, 0.3 Na_3GTP, 10 TEA, adjusted to pH 7.3 with CsOH for synaptic measures, or 125 K^+-gluconate, 10 NaCl, 1 $CaCl_2$, 2 $MgCl_2$, 10 HEPES, 2 MgATP, and 0.3 Na_3GTP, adjusted to pH 7.3 with KOH for I/V calculations. Signals were amplified with a Multiclamp 700B amplifier, recorded and stored on PC using pClamp 9 (Molecular Devices, USA). Whole-cell access resistance was 10-30MΩ. The recording electrodes were placed within the dorsolateral striatum. All the experiments were conducted in the continuous presence of picrotoxin (50 μM), added to the perfusing solution, to block contamination of the corticostriatal EPSPs with depolarizing $GABA_A$-mediated potentials. Input resistances and injected currents were monitored throughout the experiments and variations of these parameters higher than 20% led to the rejection of the experiment. For intracellular recordings, sharp electrodes were filled with 2M KCl (30–60 MΩ). Signals were recorded using an Axoclamp 2B amplifier (Molecular Devices), displayed on a separate oscilloscope, stored and analyzed on a digital system (pClamp 9, Molecular Devices). Offline analysis was performed using Clampfit (Molecular Devices, USA) and GraphPad Prism software. Glutamatergic excitatory postsynaptic potentials (EPSPs) were evoked by electrical stimulation every 10 seconds and, to induce Long-Term Potentiation (LTP), we used a high frequency stimulation (HFS) protocol, consisting of three trains of 100 Hz, 3 seconds of duration and 20 seconds of inter-train interval. During tetanic stimulation, the intensity was increased to suprathreshold levels. For the LTP protocol, at the beginning of intracellular recordings, after a stable baseline of 10 minutes, magnesium ions were omitted by switching the medium to a Mg-free Krebs solution, to increase the NMDA-mediated component of EPSP (Bagetta et al., 2012; Ghiglieri et al., 2016). This protocol is needed to induce corticostriatal LTP in SPNs. To depotentiate a previously induced LTP, a LFS

protocol of 10 minutes at a frequency of 2 Hz was delivered 10 minutes after LTP induction. Quantitative data on EPSP modifications induced by HFS protocol are expressed as a percentage of control, the latter representing the mean of responses recorded during a stable period (15–20 minutes) before the HFS. Current–voltage relationships were obtained by applying steps of current of 100 pA in both hyperpolarizing and depolarizing direction (from -400 to +200 pA) in order to measure the membrane ability to accommodate and fire in response to current steps.

Paired Student's *t* test was used to compare the pre-HFS (as mean of values at the -2nd and -4th minutes) versus post-HFS (as mean of values at the 8th and 10th minutes) protocol and post-HFS (as mean of values at the 8th and 10th minutes) versus post-LFS (as mean of the values at the 32nd and 34th minutes) protocol in the same cell population recorded from the same experimental group (paired). Unpaired Student's *t* test was used to compare post-LFS (as mean of the values at the 32nd and 34th minutes) values of the two experimental groups. The analyses were done using Prism 8.0 software (GraphPad Software).

Biochemical experiments

Contralateral and ipsilateral striatal tissues from sham-operated and 6-OHDA lesioned rats treated with Rapamycin or Vehicle, and 6-OHDA lesioned rats treated with Rapamycin plus L-DOPA or Vehicle plus L-DOPA, were sonicated in 1% SDS and boiled for 10 minutes. Aliquots (2µl) of the homogenate were used for protein determination using a Bio-Rad Protein Assay kit. Equal amounts of total proteins (15-30 µg) for each sample were loaded on precast 4-20% gradient gels (BioRad Laboratories, Hercules, California, USA). Proteins were separated by SDS-PAGE and transferred to PVDF membranes (GE Healthcare, Chicago, Illinois, USA) via the Trans Blot Turbo System (BioRad Laboratories, Hercules, California, USA). The membranes were incubated with antibodies against p44/42 MAPK (Erk1/2) (1:2000; 9102, Cell Signaling Technology-The Netherlands), and S6 Ribosomal Protein (1:1000; 2217, Cell Signaling Technology-The Netherlands). DARPP-32 (1:500; 2306, Cell Signaling Technology-The Netherlands) was used to normalize protein levels of the

analyzed striatal markers. All blots were incubated in horseradish peroxidase-coniugated secondary antibodies and target proteins visualized by ECL detection (Pierce, Rockford, Illinois, USA), followed by quantification through the "Quantity One" software (BioRad Laboratories, Hercules, California, USA). Normalized values were then averaged and used for statistical comparisons. All data are expressed as mean ± S.E.M. Statistical significance was determined by two-way ANOVA. All representative blots shown in the figures arise from cut out and pasted bands for reassembling the image. Of note, for each graph the representative bands come from the same films.

RESULTS

Abnormal Involuntary Movements induced by L-DOPA are not affected by administration of Vehicle

The possible influence of the Vehicle, in which Rapamycin is diluted, on the emergence of AIMs induced by the chronic treatment of L-DOPA was evaluated. One group of 6-OHDA rats (n=10) was chronically treated with Vehicle (2ml/kg of 5% dimethyl sulfoxide (DMSO), 15% PEG-400, and 5% Tween-20 intraperitoneal - i.p. - twice a day) for three days and then in combination with L-DOPA twice a day (10 am and 5 pm), for two weeks. Behavioral analysis of AIMs indicated that L-DOPA treatment caused the development of dyskinetic movements in both experimental groups, with comparable extent of severity (Figure S1A left panel, scores obtained in all the AIMs test sessions, L-DOPA alone vs Vehicle+L-DOPA, green circles and blue circles, respectively; Two-way ANOVA Interaction Factor, Session x Treatment Interaction, $F_{(8,\ 1184)}=1.178$, Bonferroni's post-hoc test, *ns*; right panel, total AIMs mean of Vehicle+L-DOPA group and L-DOPA alone group, unpaired Student's *t* test t=1.070, df=91, *ns*).

Recovery of LTP and loss of synaptic depotentiation induced by L-DOPA are not affected by administration of Vehicle used for rapamycin solution.

Electrophysiological recordings show that corticostriatal LTP, in agreement with previous reports (Picconi et al., 2003; Picconi et al., 2008; Cerovic et al., 2015; Ghiglieri et al., 2016), was restored in all the SPNs recorded from 6-OHDA–lesioned rats chronically treated respectively with only L-DOPA (Figure S1B left panel, green circles, paired Student's *t* test, pre-HFS vs 10 minutes post-HFS $t=4.187$, $df=12$, ##$p<0.01$, $n=6$ cells) and with the combination of Vehicle and L-DOPA (Figure S1B left panel, blue circles, paired Student's *t* test pre-HFS vs 10 minutes post-HFS, $t=8.391$, $df=13$, $p<0.001$, $n=7$ cells). We then tested if the combined treatment was able to restore a bidirectional plasticity, through the application of a LFS protocol in corticostriatal slices that expressed LTP. As hypothesized, independent of Vehicle administration, SPNs recorded from animals treated with L-DOPA were unable to reverse their synaptic potentiation (Figure S1B left panel, L-DOPA alone group, green circles, paired Student's *t* test post-HFS vs 14 minutes post-LFS, $t=1.390$, $df=11$, *ns*, $n=6$ cells; Vehicle+L-DOPA group, blue circles, paired Student's *t* test post-HFS vs 14 minutes post-LFS $t=3.752$, $df=13$, $p<0.01$, $n=7$ cells), as clearly showed by the representative EPSP amplitude traces (Figure S1B, right panel).

Rapamycin administration does not perturb corticostriatal LTP maintenance rescued by chronic L-DOPA treatment

We also evaluated the possible effects of Rapamycin+L-DOPA treatment on the synaptic potentiation. From our results the expression of the HFS-induced LTP was maintained during the whole cellular recording time (paired Student's *t* test pre-HFS vs 10 minutes post-HFS, $t=4.763$, $df=21$, $p<0.001$, $n=12$ cells) as shown in the time course of EPSP amplitude in Figure S2A.

Rapamycin administration does not perturb ERK and S6 protein levels.

Here, we analyzed the potential involvement of Rapamycin on the striatal ERK 42/44 and S6 protein levels in the total homogenates of 6-OHDA-lesioned rats treated with Rapamycin+L-DOPA, 6-OHDA-lesioned rats treated with Rapamycin, and sham-operated Rapamycin injected group, compared to their relative controls.

Two-way ANOVA showed no significant interaction in each experimental groups: 6-OHDA Vehicle+L-DOPA vs 6-OHDA Rapamycin+L-DOPA groups (lesion x treatment interaction; ERK 42: $F_{(1,18)}$=0.002809, p=0.9583; ERK 44: $F_{(1,18)}$=0.06037, p=0.8087; S6: $F_{(1,18)}$=0.0335, p=0.8568, Figure S3A-C); 6-OHDA Vehicle vs 6-OHDA Rapamycin groups (lesion x treatment interaction; ERK 42: $F_{(1,14)}$=0.0671, p=0.7994; ERK 44: $F_{(1,14)}$=0.005189, p=0.9436; S6: $F_{(1,16)}$=0.3529, p=0.5608, Figure S3D-F); sham Vehicle vs sham Rapamycin groups (SHAM lesion x treatment interaction; ERK 42: $F_{(1,14)}$=0.8621, p=0.3689; ERK 44: $F_{(1,14)}$=0.5084, p=0.4875; S6: $F_{(1,16)}$=0.0458, p=0.8332; Figure S3G-I).

FIGURE LEGENDS

Fig. S1 (A, left panel) Behavioral testing to evaluate different treatments effects in the experimental groups. 6-OHDA rats co-treated with Vehicle 1 hour before L-DOPA administration (n=10 rats, blue circles), showed the same pattern of dyskinetic behavior observed over time in animals treated with L-DOPA alone (n=21 rats, red circles). (A, right) Total AIMs (limb, axial, orolingual) score observed in Vehicle+L-DOPA group and L-DOPA alone group. (B, left panel) Time course of the HFS-induced LTP and LFS-induced depotentiation in corticostriatal SPNs. (B, right panel) Representative EPSP amplitude traces to show the recovery of the LTP in each experimental group (L-DOPA alone and Vehicle+L-DOPA). An application of LFS protocol did not induce depotentiation in either experimental groups.

Fig. S2A (left panel) Time course of the HFS-induced LTP on corticostriatal SPNs of Rapamycin+L-DOPA group. (right panel) Representative EPSP amplitude traces show the long lasting recovery of LTP.

Fig. S3 (A-C) Western blot analysis of ERK 42, ERK 44 and S6 total protein levels in contralateral and ipsilateral striata of 6-OHDA-lesioned rats treated with Vehicle+L-DOPA (n=5) or Rapamycin+L-DOPA (n=6). (D, E) ERK 42, ERK 44 total protein levels in dopamine denerveted rats treated with Vehicle (n=5) or Rapamycin (n=4) and (F) S6 levels (n=5/treatment). (G, H) ERK 42, ERK 44 total protein levels in sham-operated rats treated with Vehicle (n=4) or Rapamycin (n=5) and (I) S6 levels (n=5/treatment). All data are expressed as mean ± S.E.M. Statistical significance was determined by two-way ANOVA.
