# Supplementary figures and images for: Rapamycin, by Inhibiting mTORC1 Signaling, Prevents the Loss of Striatal Bidirectional Synaptic Plasticity in a Rat Model of L-DOPA-Induced Dyskinesia
Source: Front Aging Neurosci. 2020 Aug 11;12:230. doi: 10.3389/fnagi.2020.00230 (PMC7431470; doi:10.3389/fnagi.2020.00230)

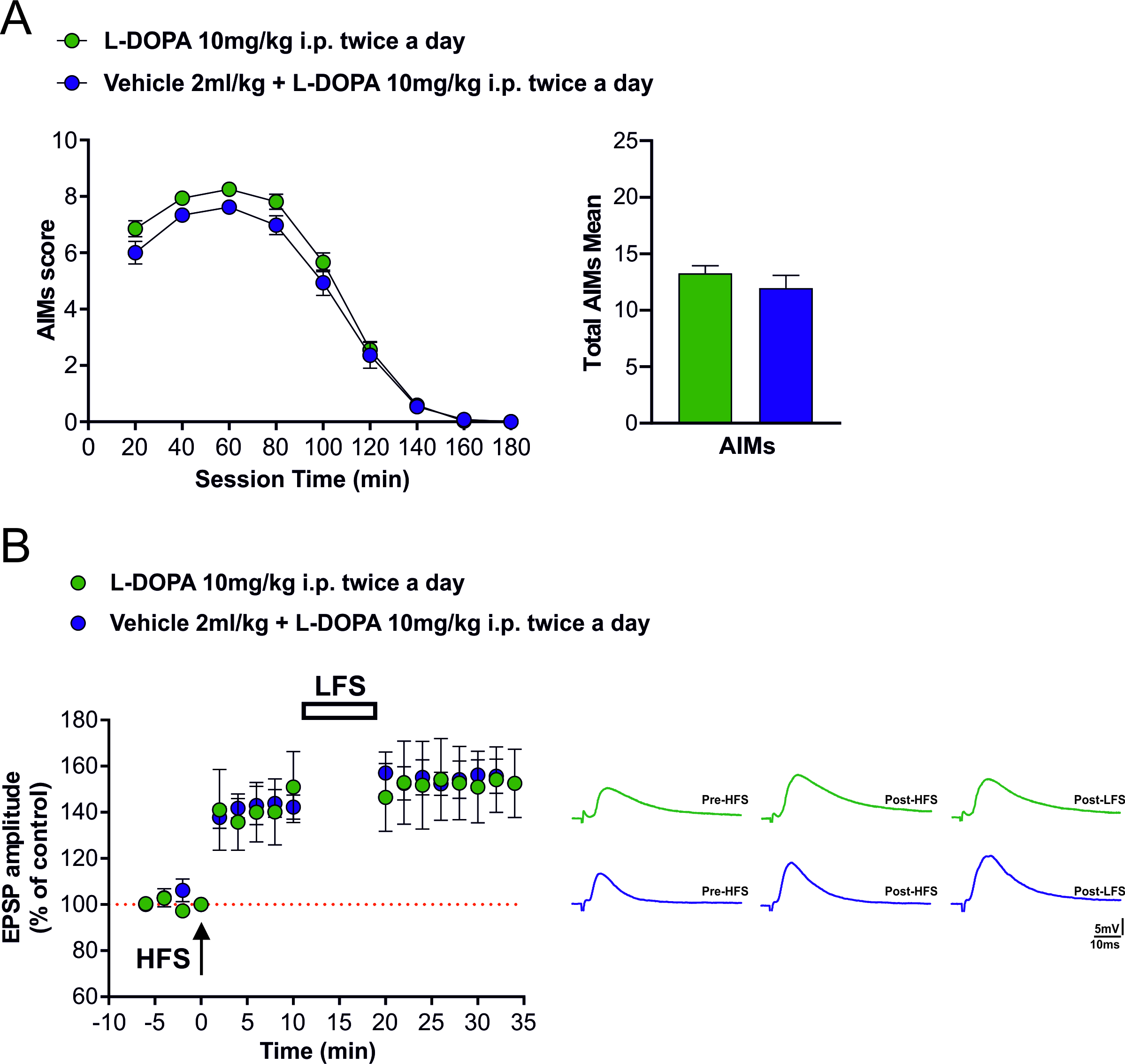

Supplement: Supplementary file 2 [file Image_1.JPEG]

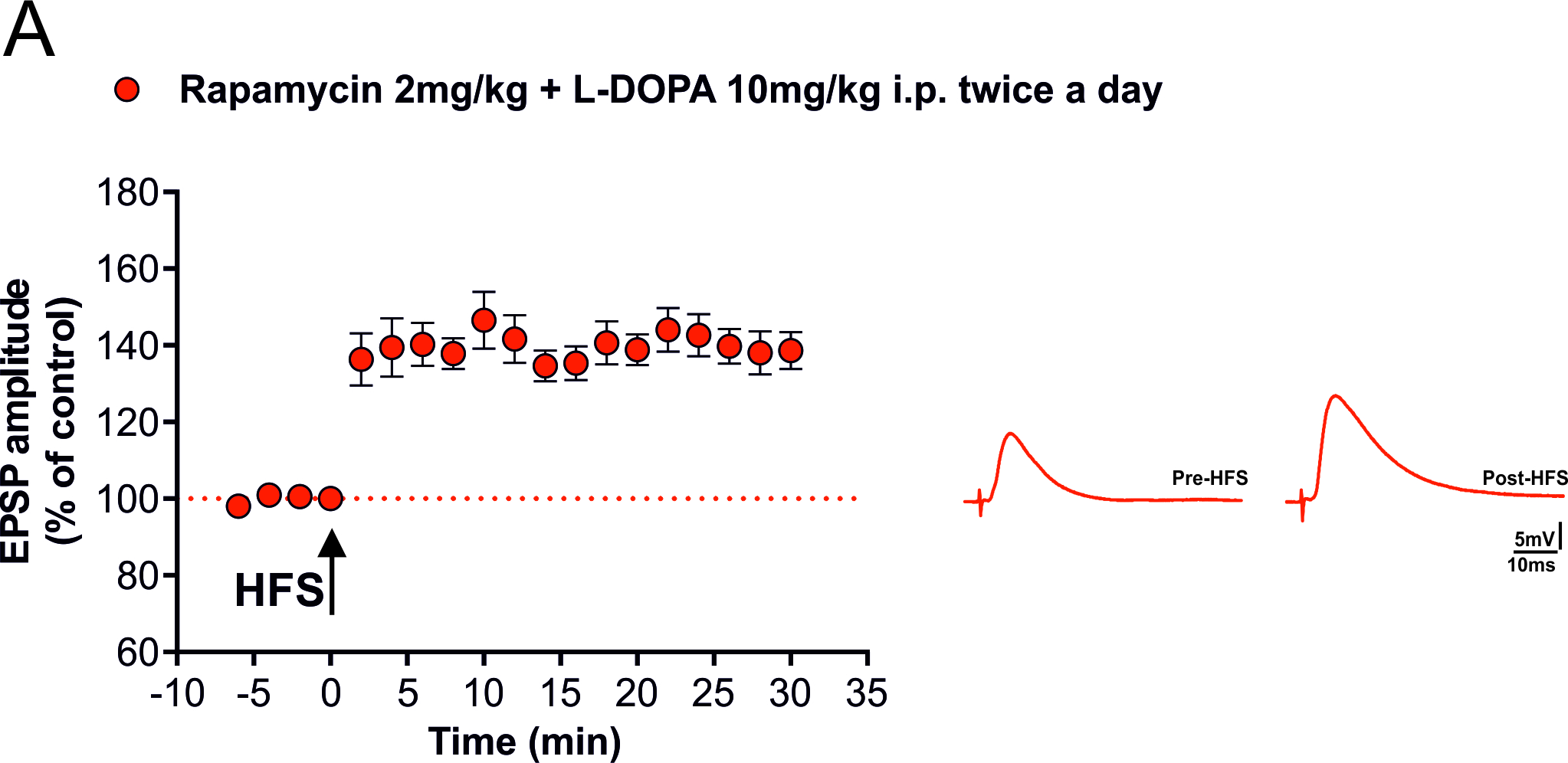

Supplement: Supplementary file 3 [file Image_2.JPEG]

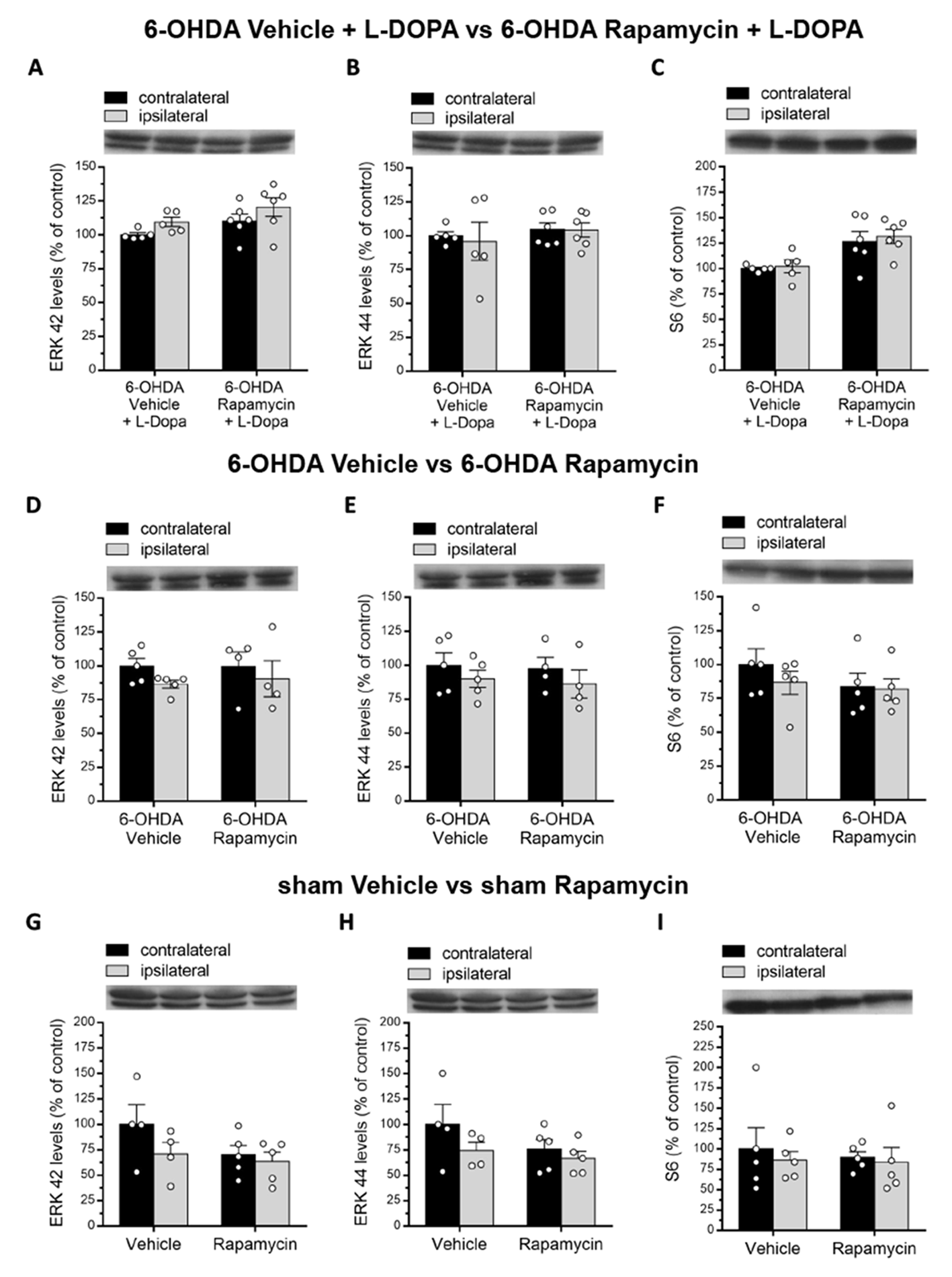

Supplement: Supplementary file 4 [file Image_3.TIF]
